# Prediction of response to GLP-1 receptor agonist therapy in Japanese patients with type 2 diabetes

**DOI:** 10.1186/1758-5996-6-110

**Published:** 2014-10-15

**Authors:** Kenjiro Imai, Tetsuro Tsujimoto, Atsushi Goto, Maki Goto, Miyako Kishimoto, Ritsuko Yamamoto-Honda, Hiroshi Noto, Hiroshi Kajio, Mitsuhiko Noda

**Affiliations:** Department of Diabetes, Endocrinology, and Metabolism, Center Hospital, National Center for Global Health and Medicine, Tokyo, Japan; Department of Diabetes Research, Diabetes Research Center, National Center for Global Health and Medicine, 1-21-1 Toyama, Shinjuku-ku, Tokyo, 162-8655 Japan

**Keywords:** Glycemic control, Glucagon-like peptide-1 agonist, Predictors of response, Preprandial blood glucose level, Liraglutide, Exenatide

## Abstract

**Background:**

Glucagon-like peptide-1 (GLP-1) receptor agonists can maintain good glycemic control in some diabetic. Here we compared the clinical characteristics and parameters reflecting glucose metabolism at the time of the initiation of GLP-1 receptor agonist therapy between patients who responded well to therapy and those who did not.

**Methods:**

The records of 43 patients with type 2 diabetes who started receiving GLP-1 receptor agonist therapy during hospitalization were retrospectively reviewed. Glucagon stimulation tests were performed, and patients were started on liraglutide or exenatide therapy. Preprandial blood glucose levels were measured on days 2 and 3 of GLP-1 receptor agonist therapy. We used the Cox proportional hazard model to compare clinical parameters between responders (HbA1c level <8% at more than 3 months after the initiation of treatment) and non-responders (HbA1c level ≥8% at more than 3 months after the initiation of treatment or a switch to insulin therapy at any time).

**Results:**

Twenty-six of the 43 patients were classified as non-responders. At baseline, mean HbA1c levels were 9.9% among responders and 9.7% among non-responders. Compared with treatment with only diet or metformin, the hazard ratio [HR] for non-response was 5.3 (95% confidence interval [CI]: 1.16-24.6, *P* = 0.03) for insulin therapy and 5.0 (95% CI: 1.13-22.16, *P* = 0.03) for sulfonylurea therapy. Compared with the lowest tertile, the HRs for non-response in the highest tertile were 3.1 (95% CI: 1.04-8.97, *P* = 0.04) for the mean preprandial blood glucose level on days 2 and 3 and 3.4 (95% CI: 1.05-11.01, *P* = 0.04) for the body mass index. The response was not significantly associated with the duration of diabetes or the glucagon stimulation test results. A receiver operating curve analysis showed that the mean preprandial blood glucose level had the highest area under the curve value (=0.72) for the prediction of non-responders.

**Conclusions:**

In patients with poorly controlled diabetes, the response to GLP-1 receptor agonist therapy was significantly associated with the treatment used before the initiation of therapy, the body mass index, and the mean preprandial blood glucose level during the 2 days after the initiation of therapy.

## Background

In patients with diabetes, the maintenance of good glycemic control is the most important method for preventing the progression of diabetes-related complications. According to the position statement of the European Association for the Study of Diabetes (EASD) and the American Diabetes Association (AHA), glucagon-like peptide-1 (GLP-1) receptor agonists, such as liraglutide and exenatide, are recommended because of their ability to maintain good glycemic control in diabetic patients without resulting in weight gain or significant hypoglycemia [1, 2]. They have also been shown to help maintain β-cell mass and function [3]. GLP-1 receptor agonist therapy has not yet been widely used in Japan [4]; however, it has recently begun to attract more attention [5, 6].

Some diabetic patients respond well to GLP-1 receptor agonist therapy and some do not. For medical and socio-economic reasons, it is important to determine methods of predicting the response to GLP-1 receptor agonists. In consideration of low risk of hypoglycemia and lesser effect on body weight gain, previous studies [7–9] have been switched from insulin therapy to GLP-1 receptor agonist therapy and certain number of the patients have been regarded effective.

Previous studies [7–11] have reported that a short history of diabetes, a high fasting serum C-peptide (CPR) level, a high stimulated CPR level at 6 min during glucagon stimulation (CPR6), and a high urinary C-peptide level at the start of treatment may predict the response to GLP-1 receptor agonist therapy in terms of reducing the blood glucose levels. Combined GLP-1 receptor agonist and insulin therapy has shown promising results in patients who are modestly obese and have a longer duration of diabetes [12]. These studies have demonstrated the importance of identifying predictors of response to treatment; however, whether other factors, such as previous antidiabetic treatment and glucose levels soon after the initiation of GLP-1 receptor agonist therapy, are capable of predicting the response to GLP-1 receptor agonist therapy remains uncertain.

Therefore, the present study investigated previous antidiabetic treatment and the preprandial blood glucose levels on days 2 and 3 of GLP-1 receptor agonist therapy as well as clinical characteristics and parameters reflecting glucose metabolism before the initiation of GLP-1 receptor agonist therapy, including the change in the serum CPR level during a glucagon stimulation test and the 24-h urinary CPR excretion (U-CPR) level. These data were used to investigate potential predictors of the response to treatment.

## Methods

### Subjects and procedures

We conducted a retrospective cohort study of patients with type 2 diabetes who were admitted to the National Center for Global Health and Medicine for the treatment of hyperglycemia between September 2009 and December 2012 and who started receiving GLP-1 receptor agonist therapy during their period of hospitalization. All the patients initially received inpatient diet therapy (the optimal caloric intake was calculated as the ideal body weight × 25), exercise therapy, and multiple insulin injection therapy to maintain their preprandial blood glucose levels at <200 mg/dL.

GLP-1 receptor agonist therapy was initiated after a glucagon stimulation test, starting with liraglutide (0.3 mg daily) or exenatide (5 μg twice daily). Insulin therapy was discontinued at the time of the initiation of GLP-1 receptor agonist therapy. A maximum of two oral hypoglycemic drugs were used at a time. The maximum glimepiride dose was 2 mg daily. Since we switched patients from insulin therapy to GLP-1 receptor agonist therapy during hospitalization, we carefully monitored the patients for glucose fluctuations.

After hospital discharge, the patients returned for follow-up visits at least every 2 months. Follow-up blood tests included liver and kidney function tests and measurements of the serum lipid level, the fasting plasma glucose level, and the HbA1c level. The medication doses were increased to the maximum dose at the discretion of the attending physician, and patients were cautioned about adverse effects such as marked anorexia, nausea, or diarrhea. The maximum dose of liraglutide was 0.9 mg/day in Japan, while that of exenatide was 10 μg twice daily. The present study was approved by the institutional review board of the National Center for Global Health and Medicine, and written informed consent was waived because of the retrospective design. This study was implemented in accordance with the provisions of the Declaration of Helsinki.

The primary objective of this study was to compare the clinical parameters at the time of the initiation of GLP-1 receptor agonist therapy between patients who had achieved an HbA1c level of <8% [1] at more than 3 months after the initiation of treatment (responders) and those who had not achieved an HbA1c level of <8% (non-responders). Non-responders also included patients who were switched to insulin therapy at any time because of insufficient glycemic control.

### Laboratory evaluations

A glucagon stimulation test was conducted after an 8-h fast. The serum CPR level was measured before glucagon injection and 6 min after the injection of 1 mg of glucagon, and the difference between these two levels was calculated (ΔCPR) [13]. Urine was collected for 24 h and stored in a refrigerator. The serum and urinary CPR levels were measured using the electro-chemiluminescence method. Fasting plasma glucose concentrations were measured using the electrode method. HbA1c levels were measured using high-pressure liquid chromatography. The HbA1c values were recorded as Japan Diabetes Society (JDS) values and were then converted to the National Glycohemoglobin Standardization Program (NGSP) values as follows: HbA1c (NGSP) =1.02 × HbA1c (JDS) +0.25% [14]. All the blood samples were assayed at a central laboratory. The preprandial blood glucose levels were measured at least 3 times a day using a self-monitoring blood glucose device (One Touch® Ultra®; Johnson and Johnson, USA). The blood glucose levels were measured before breakfast, lunch, and dinner on days 2 and 3 of GLP-1 receptor agonist therapy to determine whether the long-term response could be predicted by these values.

### Statistical analysis

The objective of this study was to identify factors that could predict the response to treatment at the time of the initiation of GLP-1 receptor agonist therapy. The non-response rate was analyzed using a time-to-event survival analysis. The person-time of the follow-up was calculated from the time of initiation of GLP-1 receptor agonist treatment until the definitive event (i.e., the achievement of an HbA1c level of ≥8% at more than 3 months after the initiation of treatment or a switch in treatment to insulin therapy) or the end of the follow-up period. The hazard ratios for the response to treatment were calculated using the Cox proportional hazards model. We selected the factors that were shown to be significantly associated with the response to treatment when evaluated using univariate Cox proportional hazards analyses (*P* < 0.05) in addition to the body mass index (BMI), the duration of diabetes, CPR6, and U-CPR, which have been reported to be related to patient outcome. The subjects were grouped into 3 groups (tertiles for continuous variables), and the long-term cumulative rate of treatment failure for each group was estimated using the Kaplan-Meier method. The assumption of proportional hazards was assessed using Schoenfeld residuals (*P* > 0.05 for all the tests).

Various cutoff points were calculated using a receiver operating characteristic (ROC) curve analysis of the area under the curve (AUC), true positives, false positives, true negatives, false negatives, sensitivity, and specificity of potential predictors of the response to GLP-1 receptor agonist therapy.

All the *P* values were two-tailed, and values less than 0.05 were considered significant. All the statistical analyses were performed using Stata statistical software (version 12.1; Stata Corp., TX, USA).

## Results

This study included 43 patients with a mean follow-up period of 131 days (maximum follow-up, 585 days). Twenty-six patients were classified as non-responders, of which three discontinued GLP-1 receptor agonist therapy within 3 months because of high blood glucose levels. Table 1 shows a comparison of the baseline characteristics of responders and non-responders using univariate Cox proportional hazards analyses.

**Table 1:** Baseline characteristics of patients*

	Responder	Non-responder	Hazard ratio	***P***value	AUC
	(N =17)	(N =26)	(95% CI)		
Male (%)	64.7	61.5	0.85 (0.39-1.89)	0.70	NA
Age (years)	57.1 ± 12.2	61.9 ± 16.1	1.01 (0.98-1.04)	0.63	0.62
Body mass index (kg/m^2^) †	29.1 ± 9.8	29.5 ± 5.5	1.04 (0.98-1.10)	0.21	0.66
Exenatide (%)	58.8	65.4	1.56 (0.67-3.63)	0.31	NA
Duration of diabetes (years)	10.6 ± 8.6	14.8 ± 10.6	1.04 (0.99-1.08)	0.051	0.62
Fasting C-peptide (ng/mL)	2.4 ± 1.5	2.2 ± 1.1	0.91 (0.60-1.38)	0.66	0.52
CPR6 (ng/mL)‡	4.0 ± 2.5	4.4 ± 2.4	1.05 (0.87-1.27)	0.60	0.57
ΔCPR (ng/mL)§	1.5 ± 1.1	2.1 ± 1.5	1.15 (0.90-1.47)	0.27	0.62
C-peptide index¶	1.8 ± 1.2	1.4 ± 0.7	0.79 (0.46-1.34)	0.37	0.55
U-CPR (μg/day)	87.2 ± 47.7	91.0 ± 71.1	1.00 (0.99-1.01)	0.82	0.45
Preprandial glucose of the previous day GLP-1 initiated (mg/dL)	162.6 ± 50.4	174.2 ± 36.9	1.00 (0.99-1.01)	0.40	0.60
Average preprandial glucose level over 2 days after the initiation of GLP-1 receptor agonist treatment (mg/dL)	140.0 ± 26.1	165.1 ± 31.8	1.01 (1.00-1.02)	0.03	0.72
HbA1c (%)	9.9 ± 1.8	9.7 ± 1.6	0.93 (0.74-1.16)	0.51	0.49
Previous antidiabetic treatment (%)					
Diet and/or metformin	47.1	11.5	1.0 (Reference)		NA
Sulfonylurea	23.5	38.5	5.0 (1.13-22.16)	0.03	NA
Insulin	29.4	50.0	5.3 (1.16-24.56)	0.03	NA
Dose of insulin (unit)	21.2 ± 5.9	26 ± 8.4	NA	NA	NA

*Values for responders and non-responders to treatment are shown as the percentage (number) or mean ± standard deviation. Hazard ratios for response to treatment were analyzed using the Cox proportional hazards model. Areas under the receiver operating characteristic curve (AUC) were compared using a logistic regression analysis.

†The body mass index is the weight in kilograms divided by the square of the height in meters.

‡Serum C-peptide level at 6 min during a glucagon stimulation test.

§Change in serum C-peptide level between baseline and 6 min during a glucagon stimulation test.

¶Fasting serum C-peptide level divided by fasting plasma glucose level.

**Figure 1:**
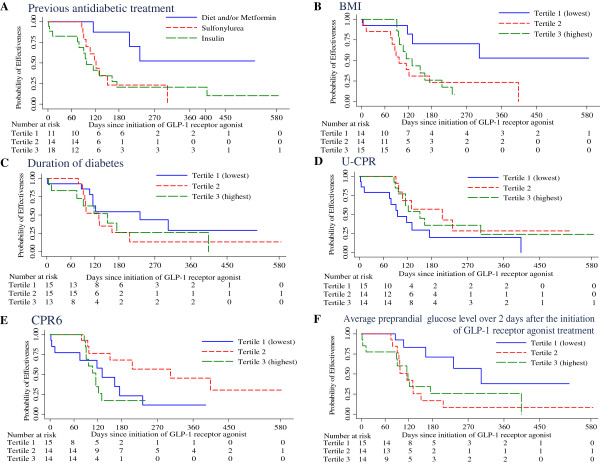
**Kaplan-Meier estimates of the cumulative incidence of non-response to treatment, up to 600 days. (A)** Previous antidiabetic treatment, **(B)** BMI: body mass index, **(C)** Duration of diabetes, **(D)** U-CPR: 24-h urinary C-peptide excretion, **(E)** CPR6: stimulated C-peptide level at 6 min during glucagon stimulation, **(F)** Average preprandial glucose level over 2 days after the initiation of GLP-1 receptor agonist treatment, GLP-1: Glucagon-like peptide-1.

There were no significant differences in sex, age, type of GLP-1 receptor agonist, or HbA1c level at the time of initiation of treatment between responders and non-responders. When a *P* level <0.05 was regarded as indicating a significant difference between groups, previous treatment other than diet or metformin was found to be a potential predictor of a non-response to GLP-1 receptor agonist therapy (Table 1). Furthermore, the BMI, duration of diabetes, and CPR6 and U-CPR levels were divided into tertiles to evaluate the effects of long-term factors on response, and the rate of treatment failure in each tertile was estimated using the Kaplan-Meier method (Figure 1).

Compared with treatment with only diet or metformin, the hazard ratio for non-response was 5.3 (95% confidence interval [CI] 1.16-24.6, *P* = 0.03) for insulin therapy and 5.0 (95% CI 1.13-22.16, *P* = 0.03) for sulfonylurea therapy (Table 2). Compared with the lowest tertile for BMI, the hazard ratio for non-response was 3.9 (95% CI 1.23-12.38, *P* = 0.02) for the middle tertile and 3.4 (95% CI 1.05-11.01, *P* = 0.04) for the highest tertile. There was no significant difference in response among tertiles according to CPR6, U-CPR, or the duration of diabetes. Compared with a duration of diabetes of <5 years, the hazard ratio for non-response was 4.1 (95% CI 0.97-17.67, *P* = 0.054) in patients with a duration of diabetes of ≥5 years.

**Table 2:** **Hazard ratios for non-response to treatment**

	N	Median (Range)	Hazard ratio (95% CI)	***P***value
BMI (kg/m^2^)	43	26.31 (19.8-52.6)		
Tertile 1 (lowest)	14	23.6 (19.8-25.0)	1.0 (Reference)	
Tertile 2	14	26.3 (25.1-30.3)	3.9 (1.24-12.37)	0.02
Tertile 3 (highest)	15	33.2 (30.4-52.6)	3.4 (1.04-11.01)	0.04
Duration of diabetes (years)	43	11 (0.1-38)		
Tertile 1 (lowest)	15	4 (0.1-6)	1.0 (Reference)	
Tertile 2	15	12 (8-17)	1.7 (0.67-4.4)	0.25
Tertile 3 (highest)	13	23 (18-38)	1.7 (0.63-4.5)	0.30
CPR6 (ng/mL)	43	3.9 (0.5-11.0)		
Tertile 1 (lowest)	15	2.0 (0.5-2.7)	1.0 (Reference)	
Tertile 2	14	4.0 (2.8-4.9)	0.4 (0.13-1.01)	0.054
Tertile 3 (highest)	14	6.7 (5.3-11.0)	1.3 (0.51-3.28)	0.59
U-CPR (μg/day)	43	71.6 (16.0-249.8)		
Tertile 1 (lowest)	15	33.7 (16.0-49.7)	1.0 (Reference)	
Tertile 2	14	73.7 (50.8-112.8)	0.4 (0.16-1.18)	0.10
Tertile 3 (highest)	14	160.0 (121.7-249.8)	0.6 (0.23-1.34)	0.19
Average preprandial glucose level over 2 days after the initiation of GLP-1 receptor agonist treatment (mg/dL)	43	149.3 (99.8-246.2)		
Tertile 1 (lowest)	15	130.3 (99.8-137.7)	1.0 (Reference)	
Tertile 2	14	149.7 (140.2-161.0)	3.5 (1.19-10.12)	0.02
Tertile 3 (highest)	14	188.7 (161.5-246.2)	3.1 (1.04-8.97)	0.04
Previous antidiabetic treatment	43			
Diet and/or metformin	11		1.0 (Reference)	
Sulfonylurea	14		5.0 (1.13-22.16)	0.03
Insulin	18		5.3 (1.16-24.56)	0.03

*Abbreviations*: *CI* confidence interval, *BMI* body mass index, *GLP-1* Glucagon-like peptide-1, *CPR* C-peptide, *CPR6* stimulated C-peptide level at 6 min during glucagon stimulation, *U-CPR* 24-h urinary C-peptide excretion.

Blood glucose levels early after the initiation of GLP-1 receptor agonist therapy were analyzed using measurements taken before 3 meals on both day 2 and day 3 of therapy. There was no significant difference in the early-morning fasting blood glucose level between responders and non-responders. Significant differences in the mean preprandial blood glucose levels were observed between the responders and the non-responders, with a hazard ratio of 1.01 per 1-mg/dL increase (95% CI, 1.00-1.02; *P* = 0.03 for trend) for day 2, 1.01 per 1-mg/dL increase (95% CI, 1.00-1.02; *P* = 0.04 for trend) for day 3, and 1.01 per 1-mg/dL increase (95% CI, 1.00-1.02; *P* = 0.03 for trend) for days 2 and 3 combined. The hazard ratio of the mean preprandial blood glucose level for day 2 and 3 combined was 1.90 per 50-mg/dL increase (95% CI, 1.07-3.39; *P* = 0.03 for trend) and 3.61 per 100-mg/dL increase (95% CI, 1.14-11.49; *P* = 0.03 for trend).

Compared with the lowest tertile for the mean preprandial blood glucose level for days 2 and 3 combined, the hazard ratio for the non-response group was 3.5 per 1-mg/dL increase (95% CI, 1.19-10.12; *P* =0.02 for trend) for the middle tertile and 3.1 per 1-mg/dL increase (95% CI, 1.04-8.97; *P* = 0.04 for trend) for the highest tertile.

The mean preprandial blood glucose level for days 2 and 3 combined was indicated by the ROC curve analysis that had the highest AUC value (AUC = 0.72; Table 1). The corresponding optimal cut-off point, at which the sum of the sensitivity and the specificity reached a maximum, was 138 mg/dL. Diagnostic parameters, including the sensitivity and specificity, depended strongly on the chosen cutoff point (Table 3).

**Table 3:** **Diagnostic measures at various cutoff points for** the **prediction of non-response to treatment**

Predictors, Cutoff point	TP/FP	TN/FN	Sensitivity	Specificity
Average preprandial glucose level over 2 days after the initiation of GLP-1 receptor agonist treatment (mg/dL)				
<120	26/12	5/0	100	29.4
<130	25/11	6/1	96.2	35.3
<140	21/7	10/5	80.8	58.8
<150	15/6	11/11	57.7	64.7
<160	10/5	12/16	38.5	70.6
<170	9/3	14/17	34.6	82.4
<180	8/1	16/18	30.8	94.1
<190	6/0	17/20	23.1	100.0
<200	4/0	17/22	15.4	100.0

*Abbreviations*: *TP* number of true positives, *FP* number of false positives, *TN* number of true negatives, *FN* number of false negatives, *GLP-1* Glucagon-like peptide-1.

## Discussion

The results of this study suggest that the treatment used before the initiation of GLP-1 receptor agonist therapy and the mean preprandial blood glucose level during the 2 days after the initiation of therapy predicted the long-term response to treatment, while the ability to secrete insulin and the duration of the diabetes history were not useful predictors. Some patients with a higher BMI and a higher CPR6 did not respond to GLP-1 receptor agonist therapy.

Pancreatic β-cell function is reduced by 20% in patients with glucose intolerance and 50% in patients with diabetes [15]. GLP-1 receptor agonist therapy has been reported to be more effective in patients with relatively high levels of insulin secretion, as determined by the 24-h urinary CPR excretion, the fasting serum CPR level, the CPR index [9], and the CPR6 [10]. In this study, however, these markers of insulin secretion were not predictors of the response to GLP-1 receptor agonist therapy.

There are two possible explanations for these results. First, our study included patients who could maintain their insulin secretion levels to some extent, while previous studies included patients with a U-CPR of less than 20 μg/day, indicating severely impaired insulin secretion [9]. The inclusion of patients treated with sulfonylureas also likely contributed to the difference in results when compared with those of a previous study [10]. Second, glycemic control is affected by both the level of insulin secretion and the degree of insulin resistance [16]. Patients with a higher BMI, which is correlated with an increased CRP6 level (r = 0.68), tended not to respond to GLP-1 receptor agonist therapy. This finding suggested that the stimulation of insulin secretion by a GLP-1 receptor agonist might be insufficient to lower the blood glucose level when insulin resistance is present. In this context, good glycemic control might have been relatively achieved among the second tertile of patients whose insulin secretion might have been modestly preserved and who might not have had insulin resistance.

Previous studies reported that liraglutide therapy is more effective in patients with a shorter duration of diabetes [8–10]. However, other studies have reported that patients with long-term diabetes may achieve better glycemic control with exenatide therapy [11]. Most of the patients in our study had a long duration of diabetes. Therefore, the duration of diabetes was not associated with efficacy when the patients were divided into tertile groups. Although the number of patients was relatively small, patients with a ≥5-year history of diabetes were less likely to achieve good glycemic control with GLP-1 receptor agonist therapy than patients with a <5-year history.

It was previously reported that patients who had been previously treated with diet and exercise achieved a greater reduction in their HbA1c levels with GLP-1 receptor agonist therapy than patients who had previously received other treatments [17]. Patients who had been previously treated with monotherapy, particularly metformin, were also reported to achieve better glycemic control with GLP-1 receptor agonist therapy than patients who had been previously treated with multiple oral hypoglycemic agents [18]. These findings are consistent with guidelines recommending the use of GLP-1 receptor agonists as second-line therapeutic agents [1] and are also consistent with our results.

Most of the patients included in the present study exhibited sustained postbreakfast hyperglycemia before the introduction of GLP-1 therapy [19]. A glycemic control of HbA1c <8.0% could be achieved only when the blood glucose levels before lunch and before dinner as well as those before breakfast were lowered [20, 21]. Accordingly, we evaluated the predictive ability of the mean preprandial blood glucose level during the 2 days after the initiation of GLP-1 receptor agonist therapy. Of note, the effectiveness of the reduction in the glucose level in response to GLP-1 receptor agonist therapy after the initiation of therapy was preserved and possessed the ability to predict a long-term improvement in glycemic control. Evaluations using blood glucose measurements performed soon after the introduction of GLP-1 receptor agonist therapy might also possess the ability to predict the achievement of more strict glycemic control, such as HbA1c <7.0%. For this purpose, more precise measurements of blood glucose, including postprandial glucose levels [22] and continuous glucose monitoring [23], might be beneficial.

### Limitations

First, this was an observational study with a small sample size, leading to wide confidence intervals for our estimates. Second, the tertile analysis may have reduced the statistical power, since it discards within-category information, especially in studies with small sample sizes. However, this analysis avoids making the assumption of a linear relation. Third, the study population consisted of patients who began receiving GLP-1 receptor agonist therapy during hospitalization for the treatment of hyperglycemia. Both of the mean HbA1c for responders (9.9%) and non-responders (9.7%) were high. Therefore, the subjects are not representative of the general type 2 diabetic population. Fourth, for patients who were already introduced insulin therapy, switching to GLP-1 receptor agonist therapy is less common. Fifth, the discontinuation of GLP-1 receptor agonist therapy was performed at the discretion of the attending physician, and there were no clearly defined criteria for discontinuation. Finally, in this study, we could not perform separate analyses according to the use of liraglutide or exenatide because of the small number of samples. Therefore, a prospective clinical study with a stricter protocol and a larger number of patients utilizing each formulation is necessary to further evaluate the effectiveness of GLP-1 receptor agonist therapy.

## Conclusions

In patients with poorly controlled diabetes, our findings suggest that patients who have received previous treatments for diabetes other than diet and exercise or metformin, who have a high BMI, and who have a high mean preprandial blood glucose level do not tend to respond well to GLP-1 receptor agonist therapy.

## Abbreviations

GLP-1: Glucagon-like peptide-1
HR: Hazard ratio
CI: Confidence interval
EASD: European Association for the Study of Diabetes
ADA: American Diabetes Association
CPR: C-peptide
CPR6: Stimulated C-peptide level at 6 min during glucagon stimulation
U-CPR: 24-h urinary C-peptide excretion
ΔCPR: Change in serum CPR between 0 min and 6 min during a glucagon stimulation test
JDS: Japan Diabetes Society
NGSP: National Glycohemoglobin Standardization Program
BMI: Body mass index
ROC: Receiver operating characteristic
AUC: Analysis of the area under the curve
TP: The number of true positives
FP: The number of false positives
TN: The number of true negatives
FN: the number of false negatives.
